# AI-assisted identification of a novel *Orthopoxvirus* inhibitor targeting F13

**DOI:** 10.1016/j.bsheal.2024.12.002

**Published:** 2025-01-05

**Authors:** Junwen Luan, Annan Ming, Wenbo Zhao, Liyuan Zhang, Leiliang Zhang

**Affiliations:** a Department of Clinical Laboratory Medicine, The First Affiliated Hospital of Shandong First Medical University & Shandong Provincial Qianfoshan Hospital, Jinan 250014, China; b Department of Pathogen Biology, School of Clinical and Basic Medical Sciences, Shandong First Medical University & Shandong Academy of Medical Sciences, Jinan 250117, China

**Keywords:** Mpox, mpox virus (MPXV), F13, Tecovirimat, Artificial intelligence (AI)

## Abstract

Treatment of mpox virus (MPXV) is crucial for public health. However, research into drugs for MPXV has fallen behind, particularly in anticipation of sudden outbreaks. This study aimed to identify new inhibitors of *Orthopoxvirus* using artificial intelligence (AI)-assisted methods. We explored AlphaFold v2.0 to simulate the F13 protein structures of MPXV, vaccinia virus (VACV), and variola virus (VARV). Utilizing MOE2019 software, we identified amino acid binding sites suitable for small molecule docking, focusing on a phosphodiesterase active site pocket in F13. Our efforts led to the identification of JCS-2022, a promising new inhibitor that exhibited docking similarities with the known anti-poxvirus drug tecovirimat. *In vitro* experiments demonstrated that JCS-2022 had a half maximal effective concentration (EC_50_) of 0.05430 μmol/L (μM), comparable to tecovirimat’s EC_50_ of 0.04794 μM. At a dosage of 1.6 μM, JCS-2022 significantly reduced VACV plaque size, indicating effective inhibition of extracellular enveloped virus (EEV) formation. Immunofluorescence analysis confirmed a reduction in VACV-induced actin tail formation. Our findings suggest that JCS-2022 is a strong candidate for development as a small molecule inhibitor against *Orthopoxvirus*, highlighting the potential of AI-assisted methods in accelerating drug discovery for infectious diseases.

## Highlights

• Virtual screening identifies JCS-2022 as a potential inhibitor for F13.
• JCS-2022 inhibits extracellular enveloped virus (EEV) production of vaccinia virus.
• JCS-2022 reduces plague size of vaccinia virus.
• JCS-2022 reduces actin tail during vaccinia virus infection.

## 1 Introduction

Since May 2022, more than 120 countries have reported over 100,000 confirmed cases of mpox. China experienced a peak in mpox cases during the summer of 2023 [1]. In 2022–2023, the global outbreak of mpox was primarily linked to the clade IIb strain. In 2024, there has been an increase in cases in the Democratic Republic of the Congo and other countries associated with clades Ib, raising significant concerns. Therefore, prevention and treatment of the mpox virus (MPXV) is of utmost importance for public health safety. The currently available inhibitor of mpox is tecovirimat (ST-246), which specifically targets the poxvirus F13 protein [2].

F13 is a critical peripheral membrane protein closely associated with forming extracellular enveloped virus (EEV) [3]. It exhibits high conservation in *Orthopoxvirus* genus, including variola virus (VARV), MPXV, and vaccinia virus (VACV), with amino acid sequence similarities exceeding 90 %. For example, the F13 amino acid sequence similarities between VACV and MPXV reach 99.5 %. ST-246 inhibits the function of the F13 protein by binding to it, thereby preventing it from carrying out its essential roles in the viral life cycle. This interference disrupts the wrapping of infectious virions and the subsequent release of EEV into the extracellular environment [4].

Recent reports have documented tecovirimat resistance in mpox patients [5], [6], [7], [8]. Therefore, the development of new inhibitors against mpox is imperative. This study aims to screen and identify a novel small molecule inhibitor targeting the F13 protein.

## 2 Materials and methods

### 2.1 F13 simulation and molecular docking

The F13 protein structures of VACV and VARV in the *Orthopoxvirus* genus were simulated using the AlphaFold v2.0 algorithm. The amino acid sequences of the F13 proteins for VACV strain Western Reserve (WR) and VARV isolate Human/India/Ind3/1967 were used as input for these simulations. Candidate compounds were downloaded from the EMBL-EBI small molecule compound database CHEMBL (https://www.ebi.ac.uk/chembl/). The Molecular Operating Environment (MOE) software 2019 (MOE 2019) was used to dock the candidate inhibitors into the binding sites of the F13 simulations. The Compute section of the MOE software was employed in this study. The “QuickPrep” command was utilized to prepare the F13 protein, followed by the “SiteFinder” command to identify the site of action. Finally, the “Dock” command facilitated virtual docking between the small molecule and F13.

### 2.2 Cells, virus, and inhibitor

HeLa and biologics standards-cercopithecus-1 (BSC-1) cells were cultured at 37 ℃ in high glucose Dulbecco's modified Eagle's medium (DMEM) supplemented with 10 % fetal bovine serum, 1 % L-glutamine, penicillin 100 U/mL, and streptomycin 100 μg/mL. VACV WR strain is cultured in HeLa cells. JCS-2022 (CHEMBL270450) is a gift from Dr. Bingming Mao. Tecovirimat (ST-246) is from MedChemExpress. dimethylsulfoxide (DMSO) (Solarbio, D8371, China) was used to prepare the stock solutions of JCS-2022 and ST-246. The stock solutions were diluted with DMEM for the cell-based experiments to create the working solution.

### 2.3 Plaque assay

BSC-1 cells were cultured in 12-well or 6-well plates until reaching about 90 % confluency. Serial 10-fold dilutions of VACV in DMEM were added to the cells. After 1 or 2 h of incubation, the inoculum was removed, and cells were washed with 1 × phosphate-buffered saline (PBS) before being covered with DMEM. Plaques were stained with 1 % crystal violet in 10 % methanol 48–72 h post-infection.

### 2.4 Cell viability

The cell viability of JCS-2022 is measured by Cell Counting Kit 8 (CCK8) assay according to the manufacturer's protocol.

### 2.5 Immunofluorescence microscopy

HeLa cells seeded onto glass coverslips were transfected with the plasmid expressing hemagglutinin (HA)-tagged MPXV F13, then washed with PBS and fixed with 4 % paraformaldehyde (PFA) in PBS buffer for 10 min at room temperature. Fixed cells were incubated with blocking solution (PBS containing 10 % normal goat serum) for 5 min at room temperature. Next, the coverslips were incubated with primary antibody against HA in a permeabilizing buffer (0.1 % Triton X-100 in PBS containing 10 % normal goat serum) for 1 h. The coverslips were washed three times with blocking solution, followed by incubation with Alexa Fluor 488-conjugated secondary antibody and Rhodamine-labeled phalloidin for 1 h at room temperature. After being washed three times with blocking solution, the coverslips were mounted with a mounting medium. The cells were imaged using an Olympus-FV12-IXCOV microscopy.

## 3 Results

### 3.1 Structure simulation of the protein complex of F13 and potential inhibitors

We used AlphaFold v2.0 to simulate the F13 protein structures of MPXV, VACV, and VARV in the *Orthopoxvirus* genus [9]. For MPXV F13, we used the simulated structure of the F13 protein from the MPXV_USA_2022_MA001 virus strain by Zheng et al. [10]. For VARV F13 and VACV F13, we obtained the amino acid sequences of F13 protein from VACV strain WR and VARV isolate Human/India/Ind3/1967, respectively, and directly simulated them using AlphaFold v2.0. The protein prediction in this research utilized the ColabFold (v1.5.5) website, an online implementation of AlphaFold2 (https://colab.research.google.com/github/sokrypton/colabfold/blob/main/Alphafold2.ipnb). F13, a monomeric protein consisting of 372 amino acids, was modeled using AlphaFold2_ptm. Template mode was set to pdb100, and MSA options included mmseqs2_uniref_env for MSA mode and unpaired_paired for pair mode. The number of recycling steps was set to 3, and the maximum relaxation iterations were capped at 200, with the remaining parameters set to default. Following the AlphaFold2 computation, five prediction models were generated and ranked by predicted Local Distance Difference test (pLDDT) scores. The top-ranked model was selected for further research. The simulated F13 protein structure with the highest pLDDT score (MPXV F13: pLDDT = 91.9, VACV F13: pLDDT = 92.0, VARV F13: pLDDT = 91.7) was selected for further study. The 3D structures of the F13 proteins of MPXV, VACV, and VARV were imported into MOE 2019 software. The “QuickPrep” command was used for preprocessing, followed by the “SiteFinder” command to search for potential amino acid binding sites for small molecule docking. A pocket including the phosphodiesterase active site amino acid was identified, and a virtual docking site was constructed using the “Dummies” command.

**Fig. 1:**
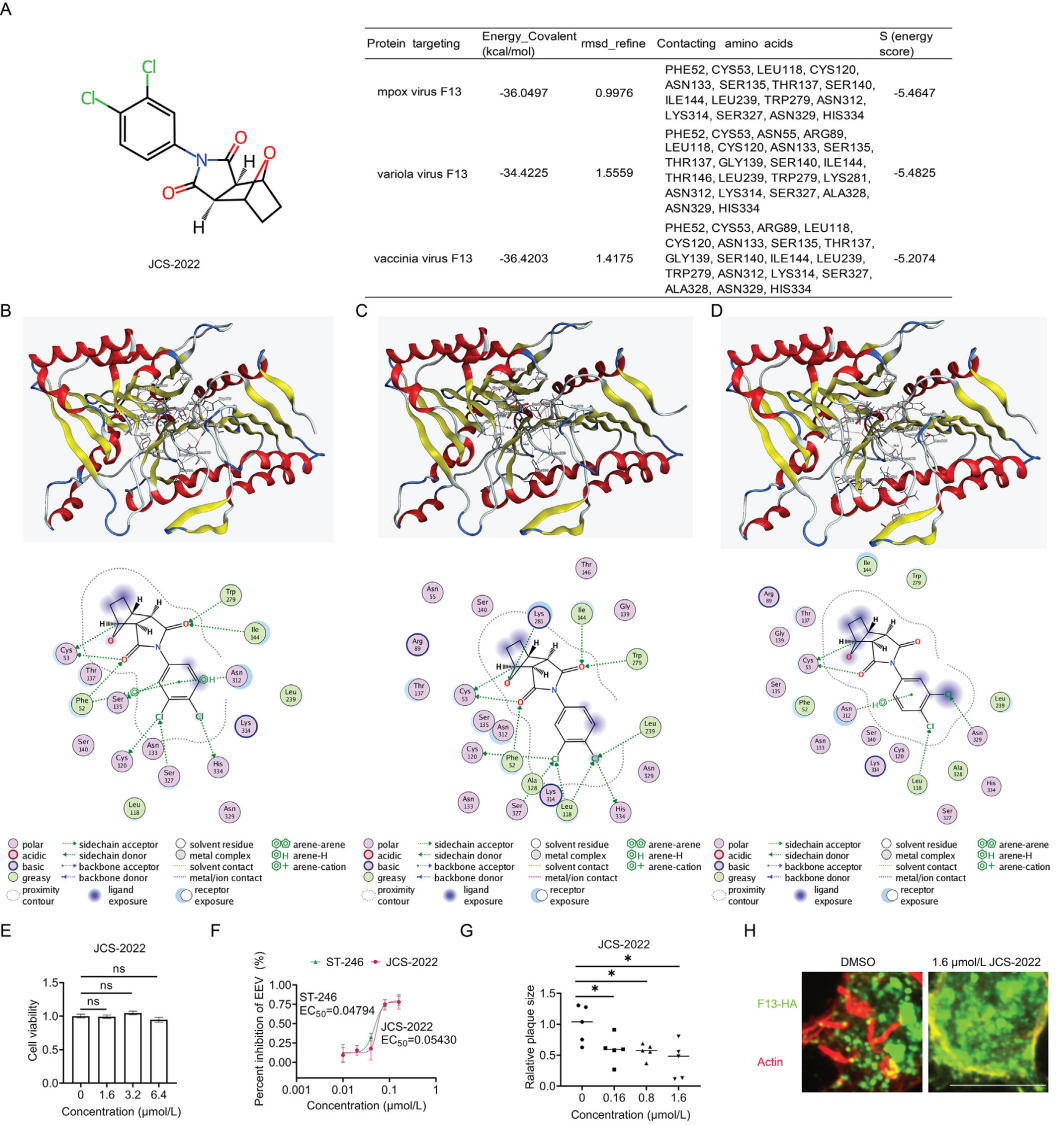
JCS-2022 inhibits VACV EEV formation by targeting F13. A) Chemical structure of JCS-2022 and the molecular docking information of JCS-2022 with F13. B) The poses with the minimum free energy of JCS-2022 along with its corresponding interactions plots within MPXV F13 protein. Different colors in F13 represent various 2D structures: red signifies α-helices, yellow indicates β-folds, and blue denotes β-turns. Amino acids within a 4.5 Å distance around JCS2022 are labeled with their names and positions. Light blue dashed lines represent interactions between molecules, with a maximum energy cutoff of −0.5 kcal/mol. C) The poses with the minimum free energy of JCS-2022 along with its corresponding interactions plots within VARV F13 protein. D) The poses with the minimum free energy of JCS-2022 along with its corresponding interactions plots within VACV F13 protein. E) HeLa cells were treated with an increased dosage of JCS-2022 for 24 h and then the cell viability was measured by CCK8 assay. ns, no significance. F) Inhibition of EEV by JCS-2022 and ST-246. HeLa cells were infected with VACV WR at a MOI of 3 for 2 h and then added increased dosages of JCS-2022 or ST-246 for another 22 h. The EEV was collected and titrated by BSC-1 cells. G) The effect of JCS-2022 with different dosages on VACV plaque size. BSC-1 cells in 12-well plates were infected with VAVC WR for 2 h and then treated with indicated dosages of JCS-2022 for 3 d. The cells were stained with crystal violet. *, *P* value < 0.05. H) JCS-2022 reduced the formation of the actin tail. HeLa cells were transfected with the plasmid expressing HA-tagged MPXV F13 for 24 h and then infected with VACV WR (MOI = 3) in the presence of 1.6 μM JCS-2022 or mock for another 24 h. The cells were stained with antibodies against HA (green). Actin tails were stained by Rhodamine-labeled phalloidin (red). Scale bar: 10 μM. Abbreviations: μM, μmol/L; VACV, vaccinia virus; EEV, extracellular enveloped virus; MPXV, mpox virus; VARV, variola virus; CCK8, Cell Counting Kit 8; WR, Western Reserve; MOI, multiplicity of infection; BSC-1, biologics standards-Cercopithecus-1; HA, hemagglutinin; EC_50_, half maximal effective concentration.

For molecular docking, firstly, we anchored our search for candidate compounds using ST-246 on the CHEMBL website (https://www.ebi.ac.uk/chembl/). The similarity threshold was set at 40 % to identify compounds with similar substructures. Redundant compounds, including ST-246 itself, were then removed. The remaining candidates were downloaded as a structure data file (SDF) file. Secondly, the SDF file was imported into MOE 2019 software for molecular docking. The “Dock” command performed virtual docking simulations at the Dummies virtual site. The docking results were compared with those of ST-246, and the three most similar candidates were selected for further analysis. Thirdly, a substructure similarity search was conducted for these three candidates, also set at 40 %, to identify additional candidates. These new candidates were then subjected to molecular docking using MOE. Ultimately, JCS-2022 (C14H11Cl2NO3; CAS:20707–31-7; CHEMBL270450) (Fig. 1A) exhibited highly similar docking results to ST-246 and was chosen as the sole candidate for experimental verification. As shown in Fig. 1B, 1C, and 1D, JCS-2022 binds to the corresponding amino acids (AA) in the phosphodiesterase active site (AA:307–334) of F13 proteins from MPXV, VARV, and VACV, suggesting that JCS-2022 may inhibit *Orthopoxvirus* such as MPXV, VARV, and VACV.

### 3.2 JCS-2022 inhibits EEV formation

Next, we conducted toxicity experiments of JCS-2022 on HeLa cells. The CCK8 assay results showed that JCS-2022 exhibited no significant cytotoxicity on HeLa cells at concentrations below 6.4 μmol/L (μM) (Fig. 1E). We then performed inhibition experiments of JCS-2022 on VACV. As shown in Fig. 1F, the half maximal effective concentration (EC_50_) values of JCS-2022 and ST-246, two small molecule inhibitors targeting VACV WR, were 0.05430 μM and 0.04794 μM, respectively. The inhibition curves of both compounds almost overlapped, especially after reaching over 75 % inhibition, which indicates that JCS-2022 has comparable inhibitory capability against *Orthopoxvirus* to ST-246, making it a promising and effective small molecule inhibitor for *Orthopoxvirus* with potential for drug development.

Furthermore, we investigated the impact of JCS-2022 on the size of VACV plaques. Experimental results demonstrated that JCS-2022 at 1.6 μM significantly reduced the size of VACV plaques (Fig. 1G). Since the production of EEV requires actin tail formation [11], we examined the effect of JCS-2022 on actin tails. Immunofluorescence experiments showed that 1.6 μM JCS-2022 reduced the actin tails in VACV-infected HeLa cells (Fig. 1H), confirming the ability of JCS-2022 to reduce EEV production.

## 4 Discussion

The small molecule compound JCS-2022 can specifically bind to the phospholipase active site of the F13 protein in *Orthopoxvirus*, thereby inhibiting the formation of EEV. Previous studies have shown that EEV production from mutant viruses lacking the *F13L* gene is strikingly lower than that from wild-type viruses [12]. Therefore, small molecules targeting F13 are preferred candidates for developing specific antiviral drugs against *Orthopoxvirus*. In this study, the AlphaFold v2.0 was used to simulate the F13 protein of MPXV, VACV, and VARV in the *Orthopoxvirus* genus. Virtual screening was then performed to simulate the molecular docking between small molecules and the F13 protein of *Orthopoxvirus*. Based on the docking results, JCS-2022 was selected as the candidate compound. Finally, experimental validation was conducted after synthesizing the candidate small molecule compound using VACV as the target. Different concentration gradients of JCS-2022 were used in virus titer experiments to determine the EC_50_ value. The results shown in Fig. 1F demonstrate that JCS-2022 and ST-246 have very similar EC_50_ values, with overlapping inhibition curves, especially after reaching 75 % inhibition. The antiviral activity experiment confirmed that JCS-2022 effectively inhibits VACV spread, making it a highly effective small molecule compound for inhibiting *Orthopoxvirus* with promising prospects and potential.

ST-246, recognized for its efficacy against *Orthopoxvirus* like MPXV, shows promising potential as an inhibitor. However, it faces several limitations that underscore the necessity for new inhibitors. Similar to many antiviral medications, prolonged use of ST-246 may prompt the emergence of viral strains resistant to its effects, underscoring the ongoing need for innovative drug development to preempt viral mutations [13], [14]. Additionally, patent protection and manufacturing capacity could challenge and restrict broad accessibility to ST-246, particularly in resource-constrained regions. The development of novel inhibitors holds promise in potentially circumventing these obstacles.

Compared to ST-246, JCS-2022 has the following advantages. First, JCS-2022 has a lower molecular weight. When using an equal amount of the compound, the mass of JCS-2022 is lower than that of ST-246. Additionally, JCS-2022 exhibits a similar inhibition curve and comparable EC_50_ value as ST-246, indicating its efficacy in inhibiting *Orthopoxvirus*. Therefore, JCS-2022 is more cost-effective than ST-246. Second, ST-246 has poor water solubility, making it difficult to formulate into liquid dosage forms. From a molecular structure perspective, both ends of JCS-2022 are polar molecules, indicating better water solubility than ST-246. In addition to being formulated into solid drugs for oral administration, JCS-2022 can also be formulated into liquid dosage forms for injection therapy, offering more flexibility in administration options.

Artificial intelligence (AI)-driven drug discovery accelerates by predicting interactions, designing compounds, and analyzing data swiftly [15]. It broadens treatments by uncovering new drug targets and repurposing medications, enhancing personalized medicine through tailored therapies. While promising cost reduction and improved outcomes, AI requires vast, high-quality datasets for practical training, hindering efficacy in drug discovery. Understanding complex biological systems and integrating AI insights into regulatory processes pose challenges, including safety and efficacy evidence. Ethical considerations like data privacy and algorithmic bias in healthcare disparities require careful navigation as AI integrates further into drug development.

Previous studies have utilized virtual screening to identify inhibitors of the F13 protein. One study highlighted natural polyphenols, identifying Demethoxycurcumin, Piceatannol, Myricetin, and Ellagic Acid as promising inhibitors of MPXV F13 [16]. Another investigation screened an in-house compound library and discovered that three compounds—Gs23, fumarate, and rupatadine fumarate—exhibit anti-pan-*Orthopoxvirus* activity *in vitro* [17]. Our research aimed to explore a broader range of synthetic and semi-synthetic molecules. This diversity in compound libraries enhances the search for effective inhibitors by allowing for the exploration of different chemical classes with varied mechanisms of action. The focus on the F13 protein in these previous studies provides an essential context for understanding the potential targeting mechanisms of various inhibitors. Our investigation of JCS-2022 opens new avenues for discussing potential novel mechanisms of action and binding, mainly through docking studies that reveal similarities to the established anti-poxvirus drug tecovirimat.

Conversely, our examination of synthetic candidates may uncover new druggable targets or improve potency through chemical modifications. The convergence of findings from different approaches underscores the importance of collaboration in this field. By sharing data and insights, we could better understand how these compounds interact with viral proteins across the *Orthopoxvirus* genus, potentially expediting the development of broad-spectrum antiviral agents. Our study significantly contributes to antiviral research by employing AI-assisted methods to identify novel compounds like JCS-2022. This work complements existing research on the efficacy of natural compounds and traditional screening techniques. This collective knowledge can help direct future research and strengthen our arsenal against *Orthopoxvirus* infections.

## 5 Conclusion

Our study identified JCS-2022 as a potential F13 inhibitor using AI-assisted virtual screening. We demonstrated that JCS-2022 inhibits EEV production of the VACV and reduces plaque size caused by the virus. As a drug presents various challenges, JCS-2022 includes safety assessments, preclinical studies, and subsequent clinical trials. Our findings indicate that JCS-2022 shows no significant toxicity at specific concentrations in HeLa cells. We plan to conduct comprehensive toxicological evaluations across various cell types to assess long-term toxicity and potential side effects. Although we have observed its antiviral effects against VACV, future research will also investigate its efficacy against MXPV and other poxviruses. Additionally, further studies are needed to thoroughly evaluate the safety, dosage, and effectiveness of JCS-2022 in mouse models of poxvirus infection to confirm its *in vivo* efficacy.
